# Humpback Whales (*Megaptera novaeangliae*) Feeding Along Migration Routes and Within Breeding Grounds—A Review

**DOI:** 10.3390/ani16172799

**Published:** 2026-09-06

**Authors:** Sarah McCulloch, Jan-Olaf Meynecke, Jasper de Bie, Madeleine J. Brasier

**Affiliations:** 1 School of Environment and Science, Griffith University, Gold Coast, QLD 4222, Australia; sarah.mcculloch2@griffithuni.edu.au; 2 Griffith Climate Change Response Program, Griffith University, Gold Coast, QLD 4222, Australia; j.debie@griffith.edu.au; 3 Whales & Climate Research Program, Griffith University, Gold Coast, QLD 4222, Australia; 4 Southern Ocean Ecosystems Program, Australian Antarctic Division, Kingston, TAS 7050, Australia; maddie.brasier@aad.gov.au; 5 Australian Antarctic Program Partnership, Institute for Marine and Antarctic Studies, Hobart, TAS 7004, Australia

**Keywords:** adaptation, behaviour, foraging, marine mammals, migration, humpback whale

## Abstract

Humpback whales traditionally fast during migration and on breeding grounds, relying on reserved energy within blubber to sustain them. However, occurrences of feeding along migration routes and within breeding grounds have been documented for several populations, suggesting an intrinsic ability to adapt to changing conditions and prey variability. This systematic quantitative literature review yielded 77 publications, demonstrating that humpback whales feed outside their polar and sub-polar feeding grounds globally. We identified that the feeding documented within this review could generally be attributed to either opportunistic/supplementary feeding, or the identification of new or expansion of current feeding grounds. Key findings included substantial feeding behaviour in productive regions with high prey availability, including off eastern Australia, South Africa, and along the coasts of North and South America. Ontogenetic drivers may also encourage younger whales to forego migration to breeding grounds and remain along migratory routes during winter to exploit seasonally available prey. The definition of this feeding behaviour lacks specificity and may lead to underrepresentation in the literature, underscoring the need to investigate its prevalence and contribution to the behavioural budgets of these populations. Our study improves current understanding of the scope of feeding away from polar and sub-polar regions and encourages future work to further understand the drivers of the behaviour.

## Simple Summary

Humpback whales have distinct feeding areas in high latitudes and breeding areas in lower latitudes, and it is believed that these behaviours generally do not overlap. However, feeding has been observed along the migratory routes of multiple humpback whale populations and within breeding areas. To summarise the occurrence of this behaviour, we assessed any research paper that discussed humpback whales feeding away from what is traditionally determined as their feeding grounds, with a total of 77 papers analysed. Results showed that humpback whales feed outside their polar feeding areas in most subpopulations, especially in highly productive regions off Australia, South Africa, and North and South America. Feeding behaviour was either believed to represent opportunistic foraging along their migration, or identification of a new or expanded feeding ground. The terminology that is used to describe this behaviour has been unclear and inconsistent, so it is likely that this widespread behaviour is underrepresented in the scientific literature. This study improves our understanding of the rate that this behaviour is occurring and provides evidence that protection measures cannot be limited to the endpoints of migration.

## 1. Introduction

Most humpback whale (*Megaptera novaeangliae*) (hereafter HW) populations migrate seasonally between their high-latitude summer feeding grounds and their low-latitude winter breeding and calving grounds [1,2], following what is known as the traditional ‘feast and famine’ migration strategy (Supplementary Material S1), with exceptions such as the population that resides in the tropical waters of the Arabian Sea [3]. HWs are capital breeders that rely on blubber stores built during their feeding season to meet their energy requirements for migration and reproduction [4,5]. Evidence for this feast and famine cycle is supported by the scarcity of sufficient prey quantities outside their polar and sub-polar feeding grounds [5,6,7], and the absence, or limited amount, of prey remains in whales hunted in low-latitude regions or on migration paths [4,5,8,9]. HWs are thought to fast throughout winter months when in the lower latitudes of their breeding grounds [10]. Deviations to the feast and famine migration exist, including individuals remaining within feeding grounds during winter [11,12], and performing feeding outside of a population’s high-latitude feeding grounds. This feeding behaviour occurs in many species of baleen whale worldwide, including fin whales *(Balaenoptera physalus)* feeding in the Tyrrhenian Sea [13], southern right whales (*Eubalaena australis*) feeding in mid-latitude waters off Argentina [14], and gray whales (*Eschrichtius robustus*) feeding continuously along migration routes [15].

Foraging away from polar feeding grounds has been recorded in multiple HW populations, along migration routes and at stopover sites in mid-latitudes [16,17,18,19]. This feeding behaviour lacks a common definition but has previously been referred to as supplementary feeding, particularly within studies from Australian waters [18,20,21], and opportunistic feeding [22,23,24]. These terms typically describe individuals supplementing their diet outside traditional polar and sub-polar feeding grounds or feeding opportunistically during migration when environmental conditions and prey availability allow. In some instances, this behaviour may be more significant than these terms suggest, meeting the energetic demands of specific individuals, cohorts, or populations when this demand is not met from high-latitude feeding grounds [21]. Additionally, studies have documented the first recorded instances of large aggregations of feeding individuals occurring outside polar or sub-polar feeding grounds. The earliest reports described HWs in waters off South Africa, where so-called “super-groups” were observed during austral spring and summer [25,26,27,28]. These super-groups were defined as aggregations of 20 or more individuals within an estimated five body lengths of the nearest whale [27]. Comparable events have since been recorded in east Australian waters, with super-groups feeding off New South Wales and Tasmania [29], which may represent supplementary feeding behaviour in this population.

Possible drivers are presumed to include (1) increasing availability of prey sources along migration routes [30], (2) shifts in species ranges as an effect of climate change [31,32], (3) limited availability of, or competition for, prey at high-latitude feeding grounds [33], and (4) an increase in population size back to pre-whaling numbers leading to animals feeding in emerging, or returning to historic, feeding grounds [34]. Given the possible breadth and variability across populations, these drivers are likely to vary spatially.

As interest in the prevalence and drivers of this behaviour continues to grow, this review aimed to examine spatial and temporal trends in HW feeding along migratory routes and within breeding grounds. We identified relevant publications through a systematic quantitative literature review, summarising records of HW feeding behaviour, investigating possible drivers, and placing this behaviour in a global context. With growing whale populations and predicted impacts of climate change, we discuss the implications of this behaviour for marine management.

## 2. Materials and Methods

**Figure 1:**
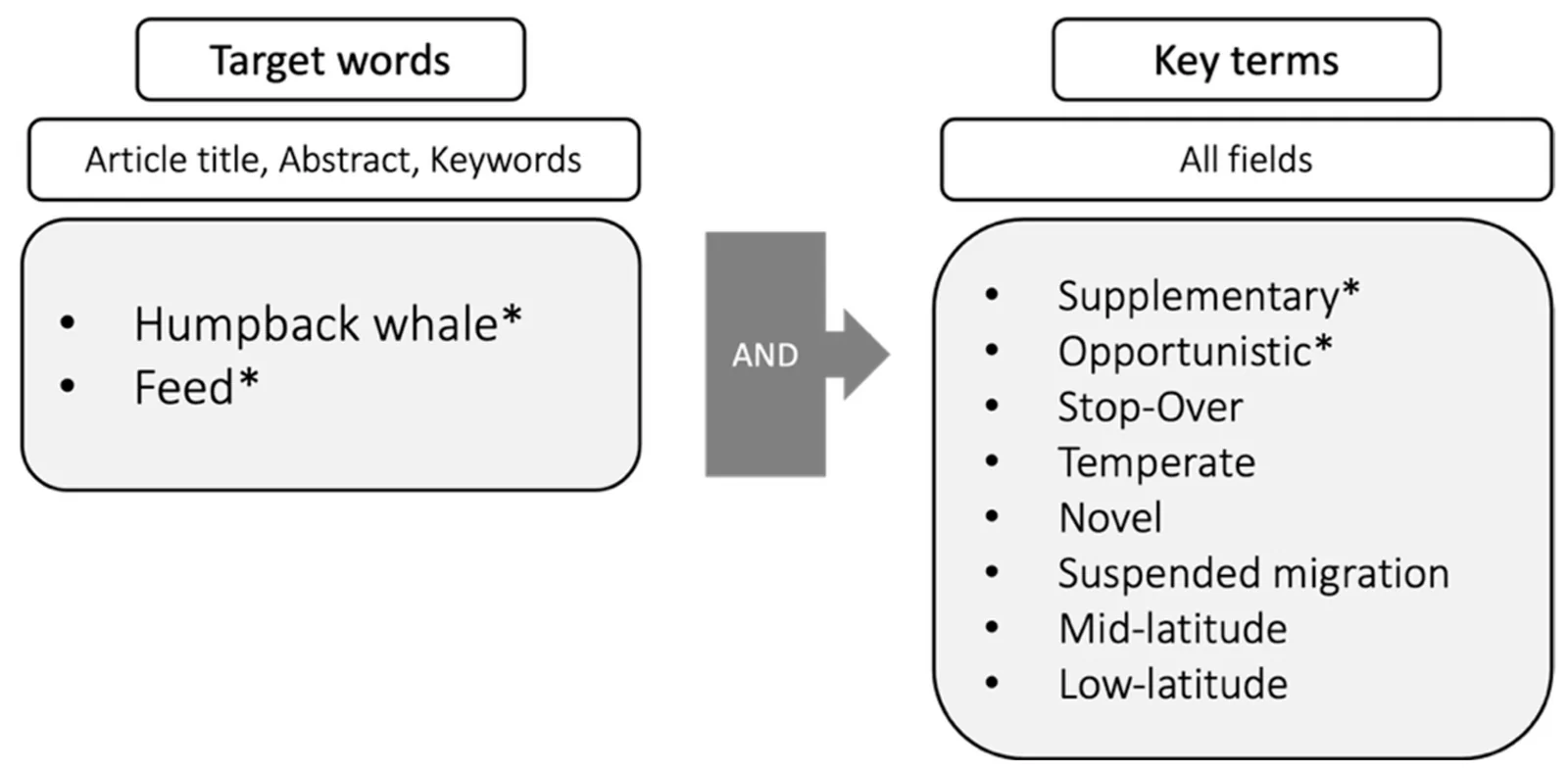
Terms used to systematically search for peer-reviewed literature pertaining to humpback whale feeding behaviour along migratory routes and in breeding grounds. Target words were combined with various key terms to search the literature. An asterisk was used as a search tool to search multiple variations of the target words.

A systematic quantitative literature review was conducted to investigate the occurrence of HWs feeding along their migratory route and within breeding grounds. The Preferred Reporting Items for Systematic Reviews and Meta-Analyses (PRISMA) flow diagram was applied for the systematic literature review [35]. The databases Scopus, ProQuest, and Web of Science were searched for publications prior to 1 January 2026. A combination of target words and key terms (Figure 1) were used as search terms, with qualifying records comprising both target words in the title or abstract, and a variation of any of the species and key terms included in the body of the text.

Commonly used terms related to feeding behaviours away from a feeding ground (e.g., supplementary feeding, opportunistic feeding, stopover, temperate, novel, suspended migration) were included as ‘Key terms’ (Figure 1). Spelling variations of these key terms were also searched (e.g., mid-latitude vs. mid latitude, supplementary vs. supplemental, etc.).

Reviews and publications not written in English were not included in the analysis. Published literature and grey literature (reports, articles, etc.) were included in the systematic review. To screen the titles and abstracts of each retrieved record, publications were excluded where they met the criteria: (1) no mention of feeding behaviour; or (2) where the feeding mentioned was within the polar or sub-polar feeding ground of that species. If it was uncertain whether a record met these criteria or not, it was included in the full-text screening stage. Next, the publications were read in full and then excluded if they did not meet the same criteria. References of the included publications were cross-checked for literature that did not include any of these key terms but still discussed feeding events outside of feeding grounds, to ensure no relevant literature was missed in the initial database searches.

Details of feeding events described in each record were documented, including if there were direct observations of feeding and how these events were recorded, number of whales feeding, age class and sex of individuals, and specific details of the feeding event(s), including if a prey species was recorded. Each record did not necessarily include information for each category, and some records included more than one answer (e.g., multiple collection and analysis methods). If details for some categories were not explicitly stated (e.g., total number of whales feeding), these were estimated based on the information provided or left as ‘unknown’. Publications were grouped into either ‘direct’ or ‘indirect’ observation of feeding behaviour. Direct observations were classified as visual records of feeding behaviour, either in boat- or land-based surveys, opportunistic sightings, or videos. Indirect observations included when feeding behaviour was inferred from records of defaecation, Area-Restricted Searching (ARS) patterns from satellite tags, feeding vocalisations, biochemical signatures representative of feeding outside of high-latitudes (e.g., stable isotope or fatty acid signatures from tissue or baleen plates), the presence of prey in the stomach, etc.

Locations of feeding events from each publication and species were mapped by the oceanic region of migration (e.g., western South Pacific, eastern Indian Ocean). Some locations were estimated to the most likely position of feeding behaviour—for example, where stranded animals were found with prey in their stomach, and researchers estimated the most probable feeding location prior to stranding. Additionally, for satellite tagging studies documenting ARS behaviours, a single position for feeding per ARS region was used (e.g., Andrews-Goff, Bestley, Gales, Laverick, Paton, Polanowski, Schmitt and Double [20]. Indirect evidence of supplementary feeding from biochemical analyses was unable to be geo-located (e.g., [36]). If multiple feeding observations along a wide geographic range were recorded, one location per region was mapped to represent these feeding events (e.g., Pirotta, Owen, Donnelly, Brasier and Harcourt [29] reported feeding events in 10 locations over ~800 km). Not every publication reported the exact number of feeding whales; some discussed the estimated number of feeding individuals, whereas others discussed the number of feeding events, pods, or percentage time spent feeding. For the purpose of this review, the number of feeding HWs was reported in distinct groups, including: 1, 2–10, 11–20, 21–100, and 100+. It was also recorded when the number of feeding whales was not stated or when it was not explicitly stated and had to be inferred from results (i.e., tables/graphs, percentage time spent feeding, etc.).

## 3. Results

**Figure 2:**
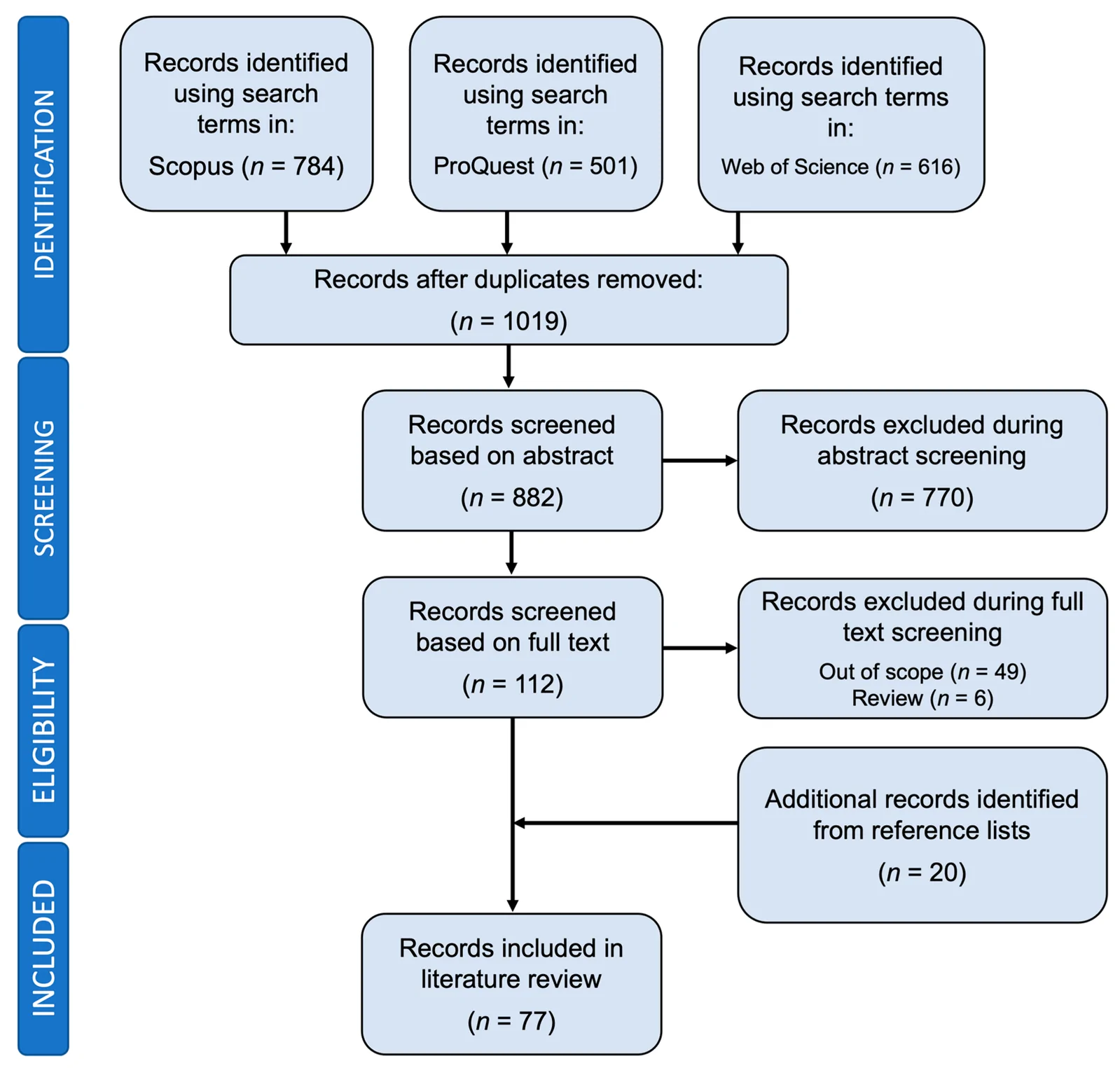
PRISMA flow diagram of the process of retrieving relevant peer-reviewed literature on supplementary feeding of baleen whales worldwide.

Of 1901 publication records retrieved from Scopus (784), ProQuest (501), and Web of Science (616), 1019 were duplicates and removed from the analysis. The remaining 882 publications were screened by title and abstract, resulting in 112 relevant publications that were analysed based on their entire text. Of these, 55 publications were excluded as they did not include information on feeding along migratory routes or in breeding grounds, including six review papers. The remaining 57 publications were deemed relevant for the current review. An additional 20 publications were identified from the reference lists of these retained papers, including historic publications and grey literature. A small number (*n* = 4) of older publications identified in reference lists were not located in online databases. Altogether, a total of 77 publications contained relevant information on HWs feeding along migration or in breeding grounds (Figure 2) (see Supplementary Material S2).

**Figure 3:**
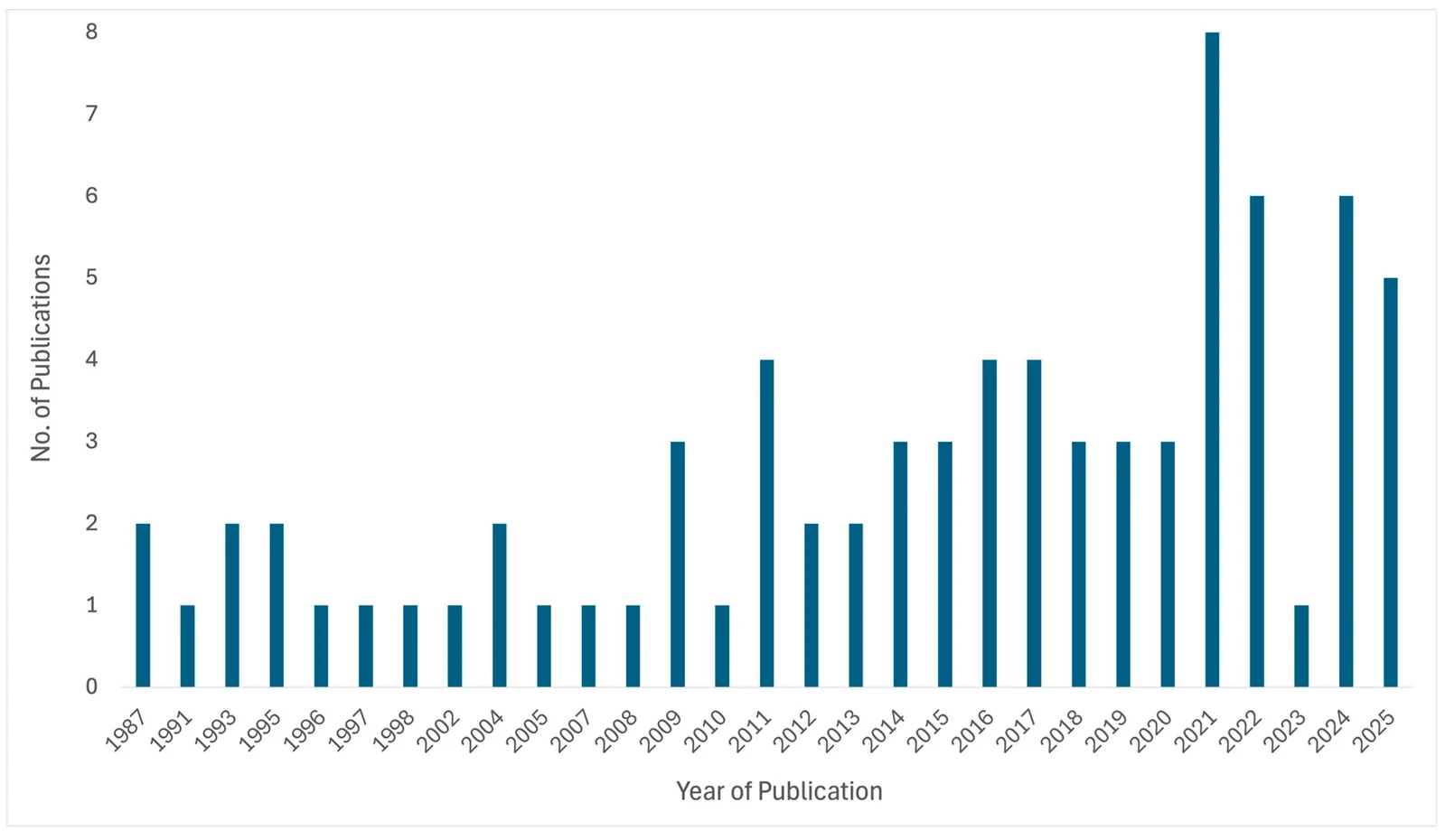
Total number of retrieved publications that discussed supplementary feeding in baleen whales between 1987 and 2025.

The years of publication ranged from 1987 to 2025 (Figure 3), with an increasing trend in the number of publications from the mid to late 2010s, and the highest number of publications in 2021 (*n* = 8). The topics discussed within the publications include feeding ecology (*n* = 42), habitat use/selection (*n* = 36), migration pathways (*n* = 19), sightings records (*n* = 17), environmental drivers (*n* = 11), species presence (*n* = 11) and distribution (*n* = 9), anthropogenic impacts (*n* = 8), prey species (*n* = 7), population structure (*n* = 5), and acoustic presence (*n* = 4). Climate change was discussed in 11 of the 77 publications, with all mentions occurring from 2011 onwards, and most (*n* = 8) between 2019 and 2025.

The number of feeding whales reported ranged from 1 to up to 200 individuals within a feeding group. In 23 publications, the number of feeding whales was not stated, and in 8 further publications, the number was not explicitly stated (e.g., estimates given, inferred from figures or other results, etc.). Where numbers were reported, 1–5 feeding whales were the most common (*n* = 25), followed by 100+ individuals (*n* = 11), 6–10 (*n* = 5), and 21–50 individuals (*n* = 6). Twenty publications reported instances of 20+ feeding individuals, and 9 of these mentioned super-groups. Prior to 2017, there were no references to super-groups found within the publications reviewed.

### 3.1. Feeding Records per Region

**Figure 4:**
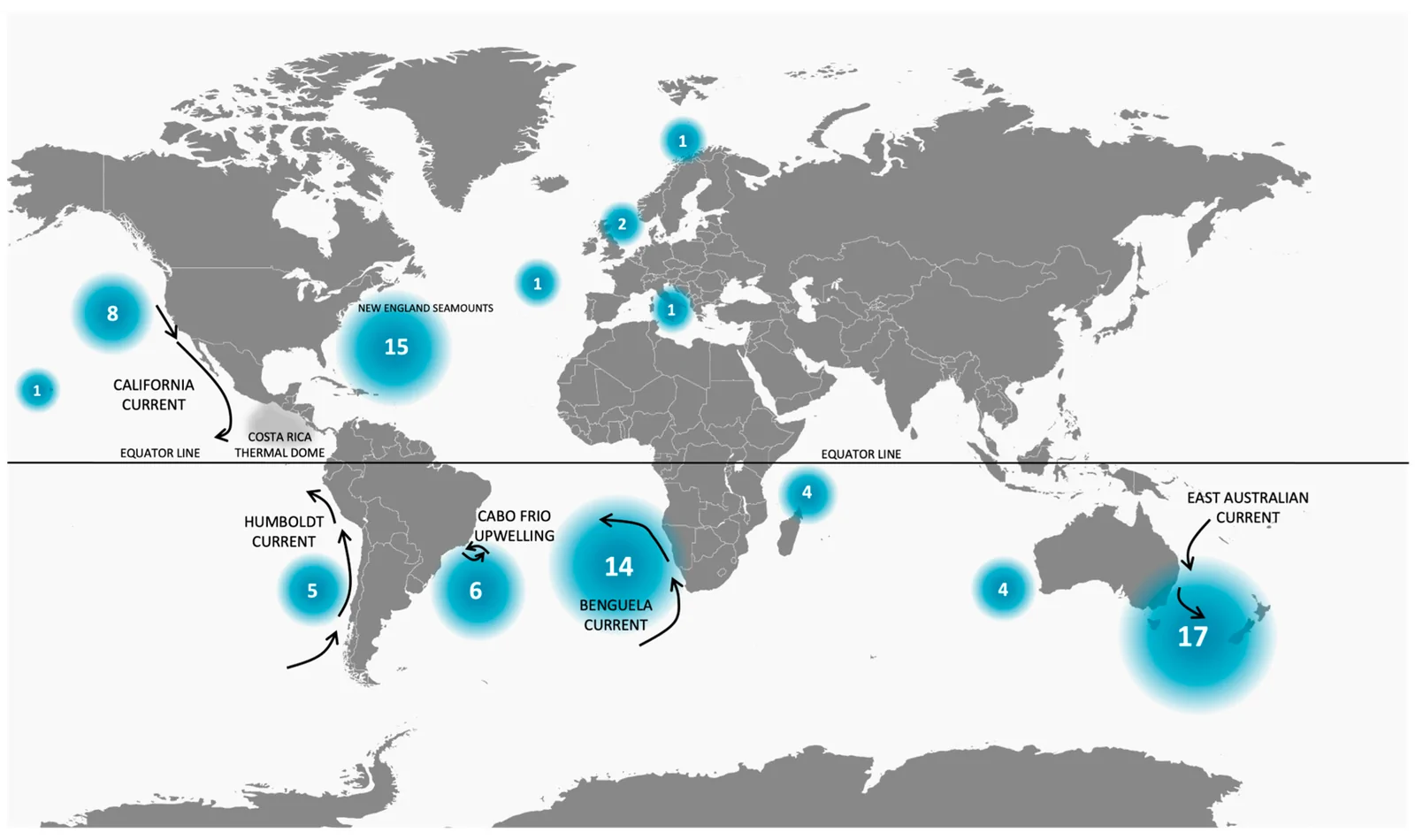
Locations (blue circles) where feeding has been reported within the literature and included in this study. Numbers indicate how many studies were conducted in each location. Arrows indicate oceanic current systems discussed within this review.

Studies were conducted in almost every ocean basin, including the South and eastern North Pacific, the Indian, and the South and North Atlantic (Figure 4). No studies from the waters off the Asian continent or Russia were identified in this review. More publications were retrieved from the Southern Hemisphere compared to the Northern Hemisphere, with 46 and 25 studies, respectively.

#### 3.1.1. Western South Pacific and Eastern Indian Ocean

**Figure 5:**
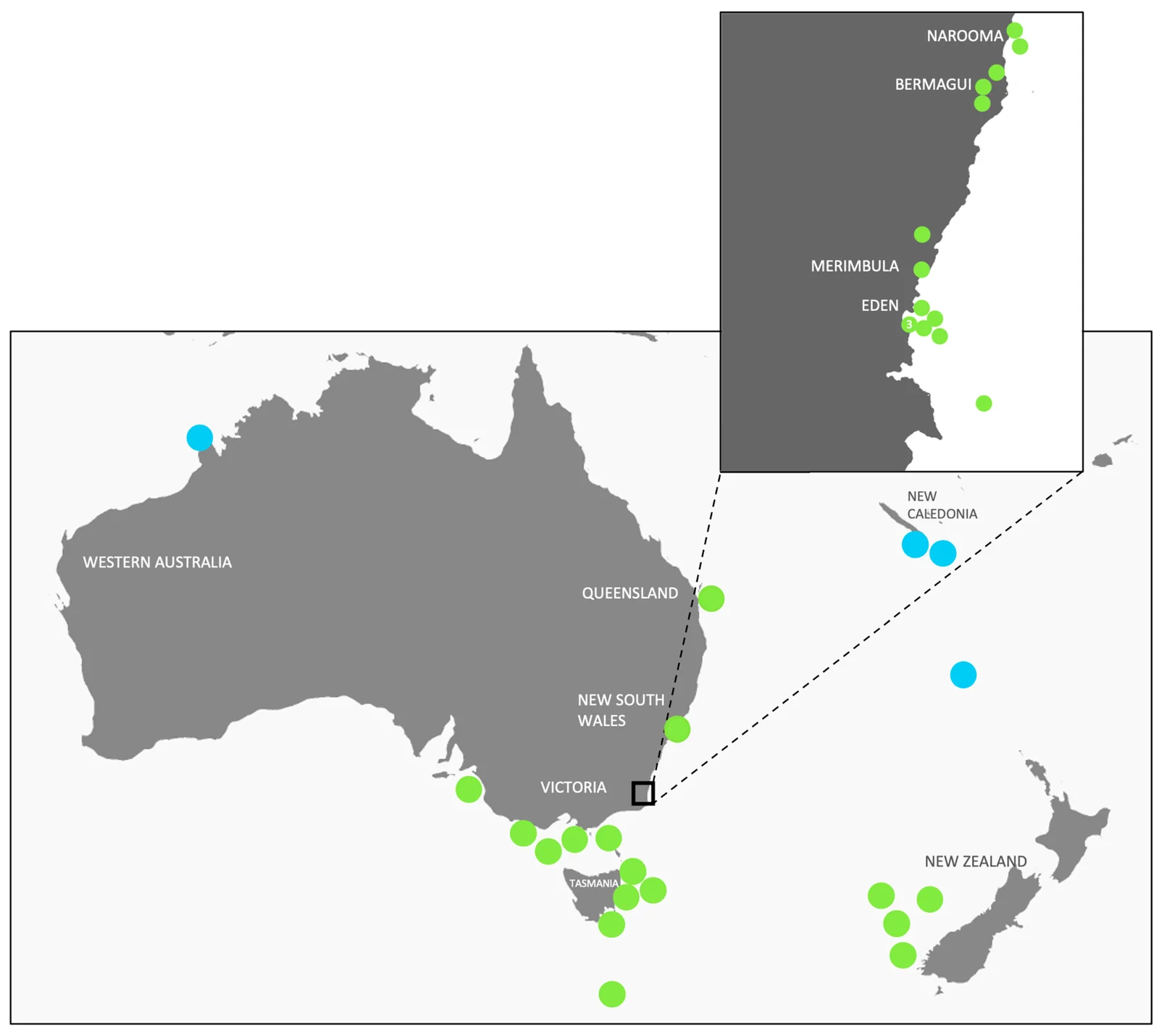
Map of feeding events recorded in the western South Pacific and eastern Indian Ocean. The magnified area shows instances of feeding from the southern New South Wales coast. Green symbols represent a direct observation of feeding (from boat or land surveys, opportunistic sightings, visual evidence, etc.) and blue represent indirect feeding inferred from the results of the study (from, e.g., records of defecation, ARS patterns from satellite tags, feeding vocalisations, stable isotope or fatty acid signatures from tissue or baleen plates, the presence of prey in the stomach, etc.). Numbers within the symbols indicate multiple studies utilising the same dataset for that region.

Between 1998 and 2024, 21 publications identified HWs feeding in the Western South Pacific and Eastern Indian Ocean, with 15 published since 2015. Most research focused on eastern Australia (*n* = 15, Figure 5), with the remainder featuring data from other Australian states (*n* = 4) and New Caledonia (*n* = 2). Foraging almost exclusively coincided with the poleward migration from August to November (*n* = 13), with an exception of an isolated record of a single adult feeding during the northward migration in July [24]. Feeding outside high-latitude feeding areas is understood to be opportunistic, with proposed ‘supplementary feeding’ regions occurring along the New South Wales and Tasmanian coastlines, where whales take advantage of seasonally available prey [18,37].

Studies in Australian waters suggest that HW feeding is driven heavily by oceanographic conditions, particularly off New South Wales. Large-scale climatic cycles, such as the El Niño–Southern Oscillation (ENSO) and Southern Annular Mode, may influence whether whales feed exclusively in the Antarctic or supplement their intake in temperate regions [36]. Regionally, shifts in the East Australian Current have been associated with feeding opportunities along the southern NSW coastline from Narooma to Green Cape [18]. These oceanographic shifts enhance regional productivity and prey availability, driving an inverse relationship between sea surface temperature and feeding behaviour [38], while intense upwelling off southern Australia is linked to aggregations of prey and elevated cetacean presence [39].

Stable isotope and radiocarbon analyses of southwest Pacific and southeast Indian Ocean HW populations revealed diverse foraging strategies, ranging from traditional Antarctic feeding to supplementary or exclusive temperate zone foraging on a mix of plankton, krill, and fish [21,37,40]. Satellite-tagging studies of HWs migrating along the east Australian coast reported ARS behaviour in temperate waters in approximately 50% (8 of 14 in 2008 and 2 of 5 tagged in 2010) of tagged individuals [20]. Consistent with this, earlier tagging studies documented increased residency times of days to weeks in areas such as eastern Bass Strait, northeast Tasmania, and southwest New Zealand, where feeding was inferred [41]. Changes to the abundance or availability of the whales’ primary food source in the Southern Ocean, Antarctic krill *(Euphausia superba)*, and a subsequent requirement to replenish energy reserves, were commonly suggested by authors as motivation for feeding [29,36,37].

In 2020, ‘super-groups’ of HWs in temperate east Australian waters were documented by whale watch operators, as well as the use of bubble-net feeding behaviour [29]. The type of prey available in temperate waters has previously been suggested as a reason for heightened feeding events. When krill is available, whales have been shown to lunge at higher rates and feed in larger group sizes (max. group size = 9, mean = 3.4) than when fish is the available prey source (max. group size = 3, mean = 1.7) [42,43]. Whilst prey species were not collected during super-group occurrences, observers during data collection suggested that both krill and schooling fish were targeted [29].

Two studies were conducted in the Oceania region, specifically New Caledonia [44,45]. Feeding behaviour was inferred from satellite telemetry tracking of HWs during their southern migration towards feeding grounds. Satellite tags deployed on 34 individuals revealed extended occupancy near seamounts [45], while data from an additional 18 individuals showed deep U-shaped dives typical of foraging [44]. The authors proposed that a combination of potential prey in the area and association with seamounts created favourable conditions for feeding.

#### 3.1.2. Western Indian Ocean

**Figure 6:**
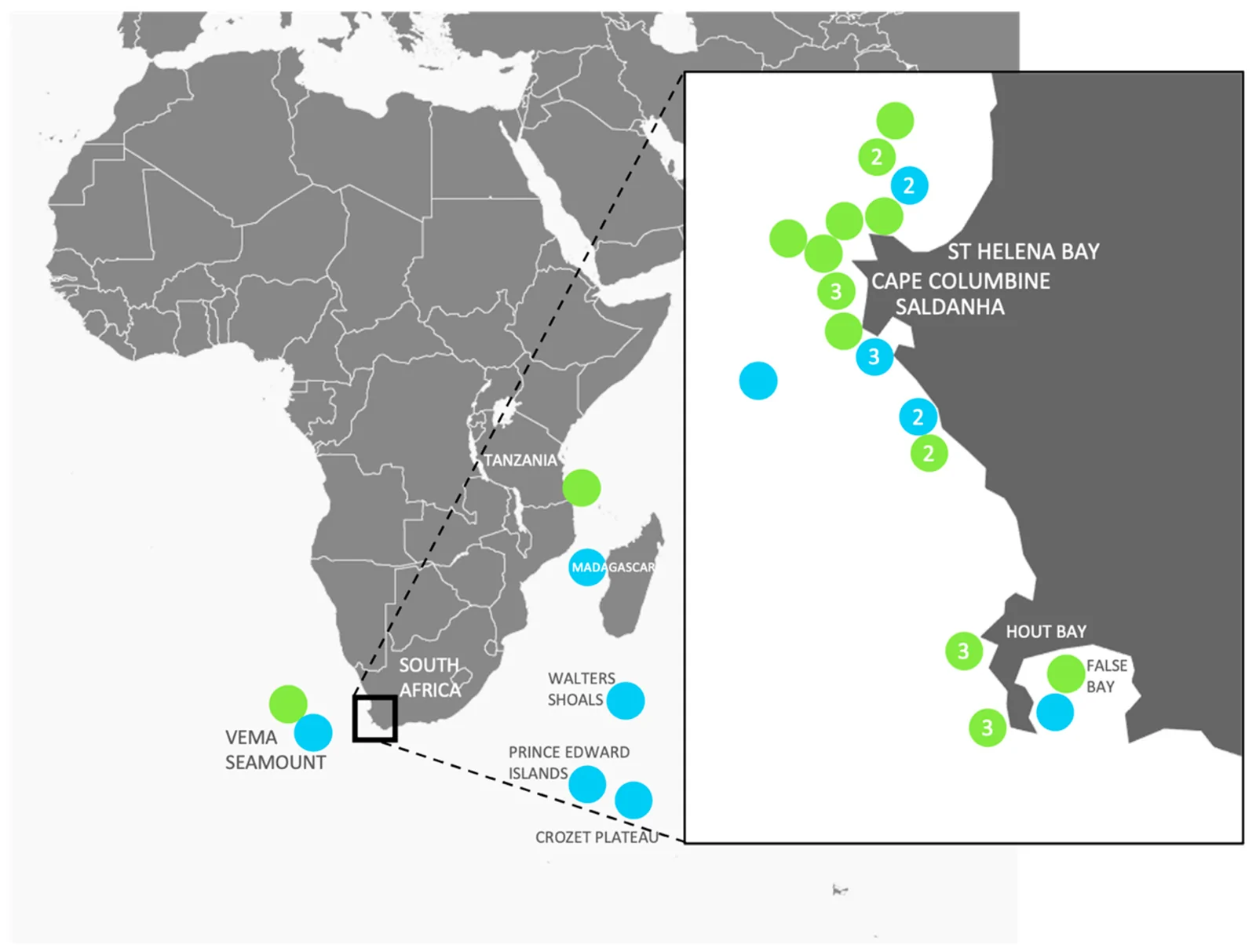
Map of feeding events recorded in the western Indian and eastern South Atlantic Ocean. The magnified area shows heightened reports of feeding off the western coast of South Africa. Green symbols represent a direct observation of feeding and blue represent indirect feeding inferred from the results of the study. Numbers within the symbols indicate multiple studies utilising the same dataset for that region.

Four publications investigated the HW population in the southwest Indian Ocean that migrates to breeding grounds off East Africa (Figure 6). In a HW breeding ground in the Mtwara region, Tanzania, whales were recorded feeding in both 2023 and 2024, and represented the first reported opportunistic feeding behaviour in this region [46]. During the post-breeding season southern migration, feeding was inferred from slow travel speeds seen during satellite tag deployments on whales along the west coast of Madagascar. In particular, feeding occurred along the west coast of Madagascar, further south near the Crozet Plateau and the Prince Edward Islands [22], as well as an area just south of Madagascar known as the Walters Shoals seamount [47,48]. Authors suggested that since these areas are associated with high chlorophyll-a (chl-a) production and phytoplankton blooms that support zooplankton growth, it was likely that feeding was opportunistic in nature [22,48].

#### 3.1.3. Eastern South Atlantic

Off western Africa, the majority of literature (12 of 14) focused on a 150 km coastal stretch between St. Helena Bay and False Bay along the South African coast, with an additional two publications identifying feeding further offshore at the Vema Seamount (Figure 6) [49,50]. Foraging intensity peaked during the post-breeding southward migration between October and December [26,27,51,52,53,54]. However, persistent feeding recorded into January and February suggests that some individuals forego the high-latitude migration entirely to remain in the mid-latitude foraging sites [28,55,56,57,58], potentially belonging to a known resident sub-stock of this population originally estimated to approximately 500 individuals [51]. While early records suggested this strategy was restricted to sub-adults, juveniles, and calves [56], recent evidence confirmed that individuals of all age classes engage in feeding [27,52].

The highly productive Southern Benguela Upwelling System drives this localised feeding behaviour [26,27,50,53,55,56]. Specifically, the bathymetry and oceanographic conditions between St. Helena Bay, Cape Columbine, and Saldanha Bay support intense upwelling, elevated chl-a production, and high euphausiid availability, while limited offshore export ensures the prey retention necessary for large-scale foraging [26,53]. The waters surrounding the Vema Seamount, west of South Africa (Figure 6), are also recognised as having high biological productivity, with two instances of HWs feeding in this region [49,50].

HW super-groups were frequently documented in the southern Benguela System. Aggregations were recorded in 2011, 2014, and 2015 [26,27], with an additional eight feeding events identified between St. Helena Bay and Hout Bay during 2015–2016 [57]. This spike in sightings may reflect population growth, prey availability, or elevated observation effort [27]. A recent update from Seyboth, Findlay, Vogel, Abras, Hurwitz, Vermeulen, Tresfon, Gridley and Elwen [28] identified super-groups occurring from August to April, with a peak during austral summer between October and January. It is still unconfirmed whether the Benguela Upwelling region constitutes a new (or previously used) feeding ground, an opportunistic foraging site, or a region of year-round residency for some individuals [28].

#### 3.1.4. Western South Atlantic

**Figure 7:**
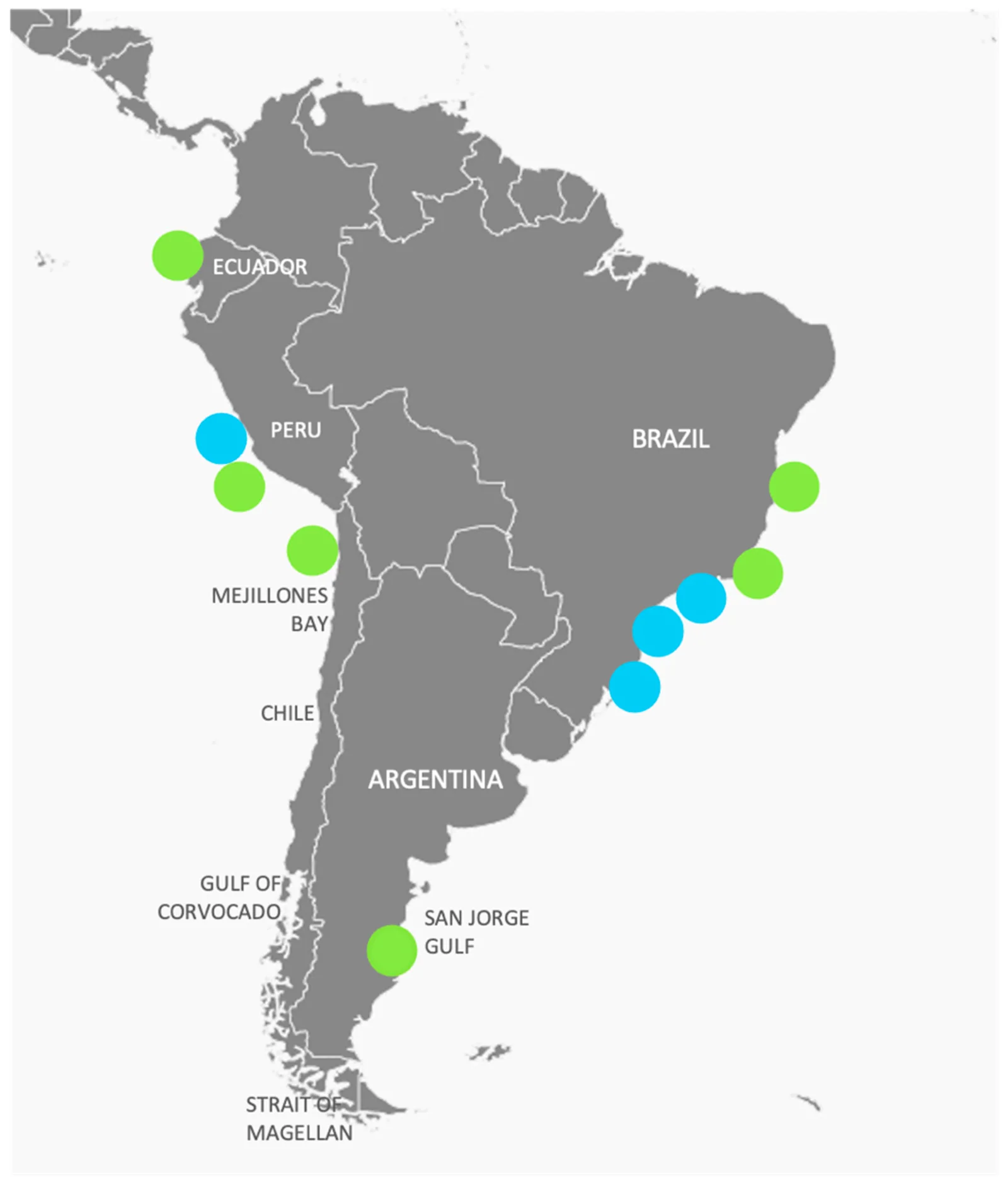
Map of feeding events recorded in the western South Atlantic and eastern South Pacific Ocean. Green symbols represent a direct observation of feeding and blue represent indirect feeding inferred from the results of the study.

Five studies documented HWs feeding in Brazilian waters [17,59,60,61,62] (Figure 7). Evidence from necropsies of stranded individuals, stable isotope analysis of skin samples, and opportunistic observations suggested that foraging within this breeding and migratory area is undertaken predominantly by yearlings and juvenile humpback whales [59,60,61]. Authors suggested that these juveniles are less capable of accruing fat reserves and thus forage during their migration to replenish their energy reserves. Additionally, the presence of the Cabo Frio upwelling system has been suggested to produce high biological productivity and provide foraging opportunities in waters off Brazil [17]. In waters off Argentina, two adults and one juvenile HW spent up to 10 days feeding in the regions of the San Jorge Gulf, Patagonia, during austral winter and spring, foregoing migration to traditional breeding habitats [63]. Feeding along the South American coastline is suggested to be opportunistic, with feeding occurring both before, during, and after the breeding season [17,59,61].

#### 3.1.5. Eastern South Pacific

In the eastern South Pacific, mid-latitude HW feeding behaviour has been documented across Ecuador and Chile [64,65] and Peru [23,66,67] (Figure 7). Within their breeding grounds, extending between Costa Rica and northern Peru, multiple feeding events have been recorded. These include observations of lunge-feeding pairs during the peak breeding season in Ecuador [64], and satellite-tracked individuals in northern Peru timing their southward migration to exploit highly productive upwelling zones [23]. This included a mother–calf pair foraging for up to four days [66]. HWs may also use Peruvian waters for feeding during their austral summer feeding season, with two individuals observed off southern Peru for at least three days during February displaying behaviour consistent with feeding [67].

In Mejilones Bay, Chile (Figure 7), two HWs were observed feeding during austral autumn, with authors suggesting individuals over summer in mid-latitude areas. The recapture of the same individual feeding during austral autumn in the consecutive year suggested that some whales may hold site fidelity to this region [64]. Prolonged upwelling from the Humboldt Current System fuels year-round primary production in the region (Figure 4), with a distinct upwelling cell adjacent to Mejilones Bay [64]. Slightly further north off northern Patagonia, Hucke-Gaete, Haro, Torres-Florez, Montecinos, Viddi, Bedriñana-Romano, Nery and Ruiz [65] reported that HWs have likely used waters around the Gulf of Corcovado as a mid-latitude feeding ground since the early 1990s. Observations between February and April, coinciding with this population’s traditional Antarctic summer feeding season, suggest this area may represent a segregated feeding group [65].

#### 3.1.6. Eastern and Central North Pacific

**Figure 8:**
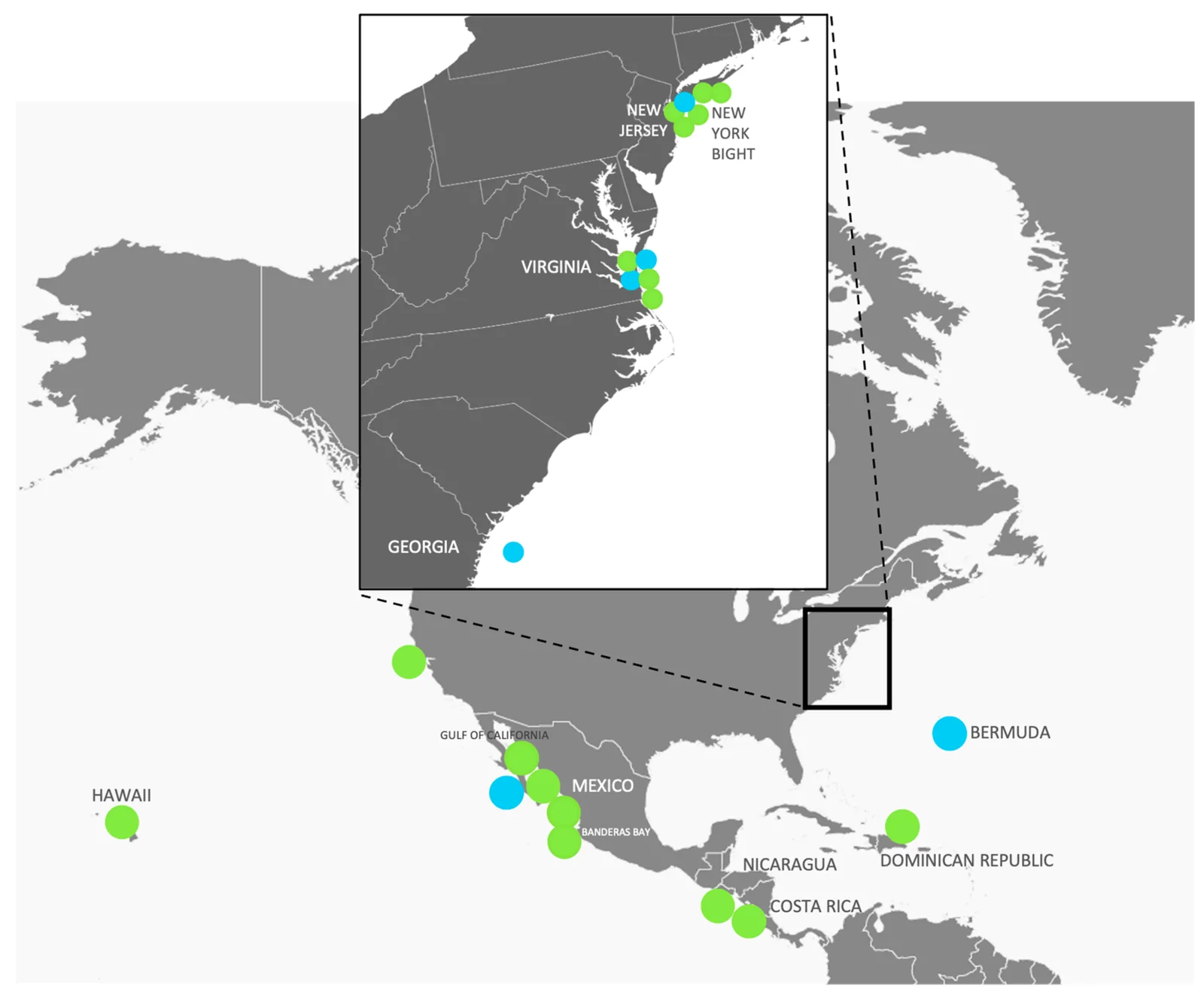
Map of feeding events recorded in the eastern North Pacific and western North Atlantic Ocean. The magnified area shows instances of feeding from the New York Bight, the coastal waters between Long Island and New Jersey. Green symbols represent a direct observation of feeding and blue represent indirect feeding inferred from the results of the study.

The population of HWs migrating through the eastern North Pacific frequently feeds opportunistically along its migratory route between breeding grounds in Central America and feeding grounds off Washington, Oregon, and California [68,69,70,71,72,73,74] (Figure 8). Studies have documented feeding within breeding grounds, including the first evidence of feeding in Nicaragua, occurring in 17.5% of sightings [68]. In Costa Rica, the population’s most southerly breeding ground, a mother with a dependent calf was observed feeding during the winter breeding season [73]. Similarly, in the Central Pacific, rare opportunistic feeding on bait balls by an adult and a juvenile was documented during the peak winter breeding season in Maui, Hawaii [75]. The authors noted that such behaviour, while uncommon, may help younger individuals replenish critical energy reserves.

Within Mexican waters, opportunistic feeding has been recorded during the boreal winter in Banderas Bay, where cooler sea surface temperatures associated with La Niña cycles create localised phytoplankton blooms [69,72]. From Baja California to the Gulf of California, the Baja California Frontal System plays a key role in structuring oceanographic conditions that promote elevated primary and secondary productivity [71]. This system is suggested to influence feeding in Magdalena Bay, where a satellite-tagged whale spent nine days in the area exhibiting ARS behaviour toward the end of its breeding season [71]. Opportunistic feeding has also been reported in the Gulf of California, including observations of a single whale bubble-net feeding in the region [70]. This region has also been proposed as a feeding ground rather than solely a calving area, with a potentially resident population of HWs in the Gulf of California appearing to feed locally year-round [74]. Along the Mexican coast, productivity is further influenced by variability associated with ENSO, which affects prey availability in the region [69,70,72,76]. Likewise, in Central America, the tropical Costa Rican Thermocline Dome brings cold, nutrient-rich upwelled waters to the surface, creating productive conditions that support feeding opportunities [68].

Further north, closer to the recognised feeding grounds of this HW population, sightings of whales in the San Francisco region have increased, with observations occurring year-round, and predominantly during spring and summer [77]. Prior to 2016, sightings in this area were considered unusual. However, the authors have suggested that the species’ foraging flexibility and capacity to shift prey sources between krill and schooling fish may have enabled this region to function as a new foraging ground.

#### 3.1.7. Western North Atlantic

Within the waters of the Western North Atlantic, publications recording feeding HWs were identified in two main regions, namely the New York Bight (NYB, *n* = 7) and the Mid-Atlantic Coast (*n* = 5, particularly centred around Virginia). However, feeding behaviour was also described in breeding grounds (*n* = 2) and the North-Atlantic Ridge (*n* = 1).

In the NYB, the coastal waters between Long Island and New Jersey (Figure 8), feeding behaviour by HWs was predominantly recorded during summer months when individuals of this northwest Atlantic population are typically in high latitudes for their feeding season [78,79,80,81]. Feeding behaviour was also observed in this area during boreal spring and autumn, likely by whales on migration to and from feeding grounds [82,83]. Authors suggested that shifting prey availabilities are likely linked to increased foraging activity in these mid-latitude regions [79,82], where whales may be finding abnormally high prey densities that support feeding opportunities [80]. Increasing abundance of prey species such as Atlantic menhaden, driven by climate-related factors and fishery regulations, may be contributing to the rise in HW numbers in nearshore waters of the NYB [84]. The waters of the NYB likely represent a southward extension of the current closest primary feeding grounds, the Gulf of Maine [82], with HWs of all ages regularly foraging in the region since 2011 [78,79,81]. However, some authors suggest this region may also be being used as a supplementary feeding area on migration to and from feeding areas [80,83].

Off the United States mid-Atlantic states, including New Jersey to North Carolina (Figure 8), HWs were observed feeding during boreal winter months, when migration to breeding grounds is normally observed, suggesting that these individuals did not complete their migration and instead fed along their migratory routes. All publications from the mid-Atlantic states reported evidence of only, or predominantly, feeding by juveniles [85,86,87,88,89]. Instead of migrating south to recognised breeding grounds, sexually immature individuals may adopt an adaptive strategy of remaining at mid-latitudes to replenish energy reserves in productive areas [88].

Feeding activity was reported near the breeding grounds of the northwest Atlantic HW population. Observations of five individuals producing bubble clouds and performing subsurface lunges in Samana Bay, Dominican Republic, were attributed to high productivity in the area [90]. It is proposed that whales pass Bermuda during spring on their northern migration to feeding grounds. Observations of two defecating HWs off Bermuda in the 1980s coincided with a zooplankton bloom, indicating that migrating individuals utilise this mid-latitude site for opportunistic foraging [91]. Further east, evidence from whaling logbooks revealed HW presence along the North Atlantic Ridge during late boreal spring and summer, suggesting that the area might have been used, at least historically, as a mid-latitude feeding ground [92].

#### 3.1.8. Eastern North Atlantic (Mediterranean Sea, North Sea)

**Figure 9:**
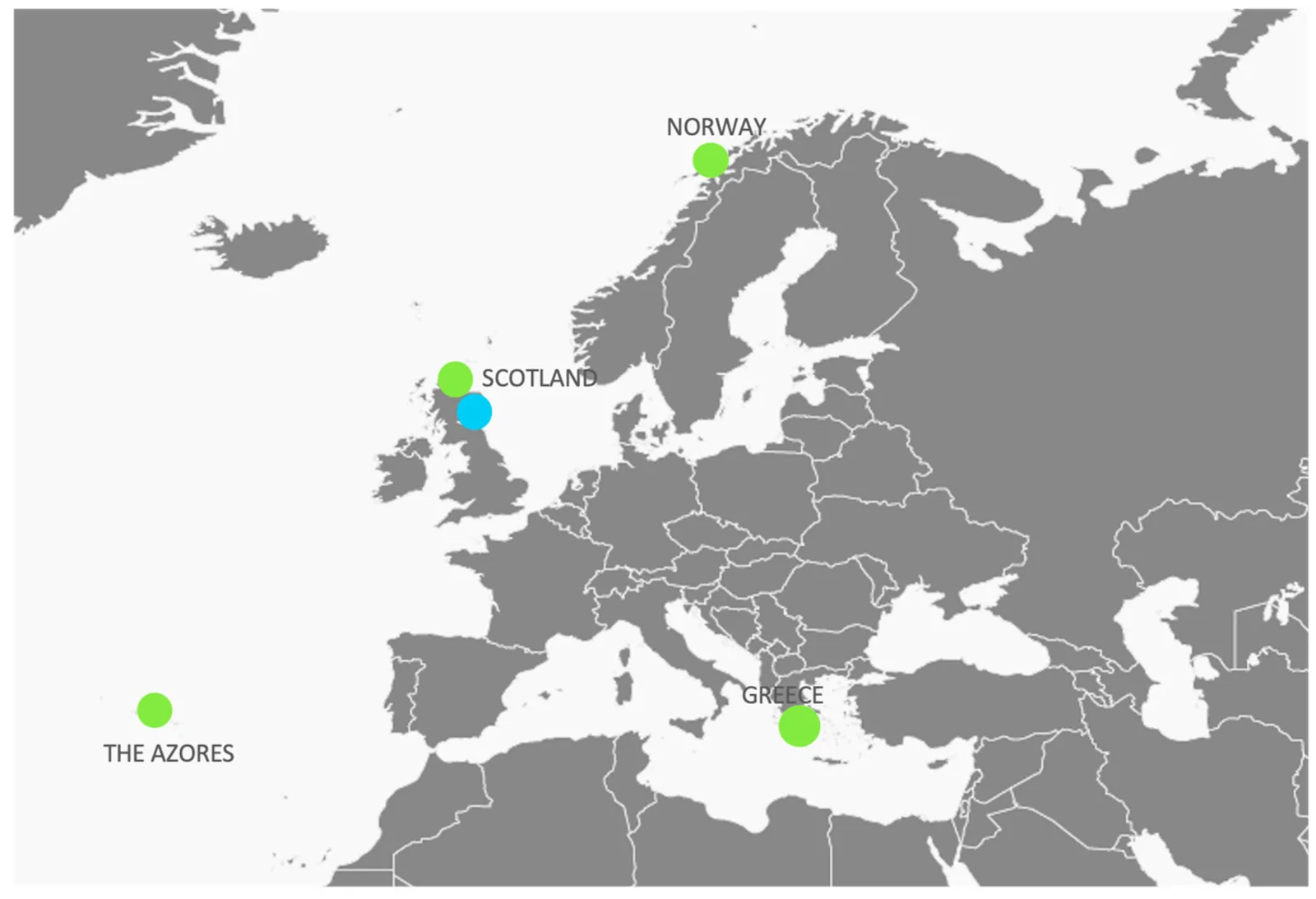
Map of feeding events recorded in the eastern and mid-north Atlantic Ocean. The magnified area shows feeding events reported from the Azores. Green symbols represent a direct observation of feeding and blue represent indirect feeding inferred from the results of the study.

Feeding behaviour by HWs has been sporadically reported in the eastern North Atlantic, as well as in the Mediterranean, North Sea and Norwegian Sea (Figure 9). In waters off Scotland, HWs were observed repeatedly feeding in multiple inlets along the eastern coast, in both boreal autumn [93,94] and winter [94] likely en route to their breeding grounds. Bubble net feeding was observed in the waters of the Moray Firth, which are highly productive and provide a predictable source of prey [93]. Feeding behaviour of a humpback whale was also observed frequently for up to one month off southern Greece [95]. This individual was likely a juvenile from the north-east Atlantic population that may have entered the Mediterranean Sea in search of food or on its first migration, leading it to migrate to novel areas [95]. In the Norwegian Sea, off the coast of Norway, HWs exhibited opportunistic feeding within mixed-species aggregations, where they were observed lunging through herring bait balls brought to the surface by killer whales. Observed during the boreal winter, this strategy was suggested to minimise energetic costs by exploiting predictable prey concentrations generated by co-occurring predators [96].

The Azores have been identified as an important mid-latitude foraging area for multiple baleen whale species (Figure 9). HWs, among other species, have been found to time their arrival in the region with the onset of the spring phytoplankton bloom [19]. Habitat selection by HWs in the region was strongly associated with elevated chl-a concentrations, with feeding comprising 77% of the behavioural budget [19].

### 3.2. Type and Quality of Data Collection

Most studies (*n* = 47) utilised multiple methods of data collection (Table 1), with the most common study method being dedicated observational boat-based surveys (*n* = 36). Publications that were intentionally investigating feeding behaviour (*n* = 41) outnumbered those that recorded feeding behaviour whilst researching other topics (*n* = 36). Most publications that were not dedicated feeding studies were investigating habitat use or species presence, and individuals in the study performed feeding.

Direct observations of individuals feeding were recorded in 52 of 77 studies. In studies without a direct observation (*n* = 25), feeding was identified by combinations of either observation of defaecation (*n* = 8), analysis of stomach contents (*n* = 6), or indirectly through other methods (*n* = 24); the latter included a combination of ARS patterns from satellite tags, recorded feeding vocalisations, correspondence between whale aggregation areas and high productivity, observation of feeding-like dive behaviour, other predatory species in the area, isotopic evidence in blubber, skin and baleen samples, and extended stays at stopover sites along migration. Observations of feeding events were predominantly recorded by dedicated boat surveys (*n* = 28), opportunistic surveys (*n* = 18), surveys from land (*n* = 8), aerial (*n* = 7) or UAV (*n* = 5) observations, or opportunistic sightings (*n* = 3).

Prey species were identified in 31 studies. Two publications recorded new prey species for humpback whales, including a sergestid shrimp (*Pisos petrunkevitch*) [59] and the Aviu shrimp (*Acetes americanus)* [60], consumed by humpback whales in southern Brazil. A full list of prey species can be found in Supplementary Material S3.

## 4. Discussion

Our systematic quantitative literature review analysed feeding behaviour occurring in the breeding grounds and along migratory routes of HW populations worldwide. This behaviour was identified to be a widespread, global phenomenon. Several locations presented higher numbers of publications reporting feeding events, including in waters off the eastern United States (the Mid-Atlantic states and NYB), off western South Africa (St. Helena to Hout Bay), and Australia (southern NSW). The feeding documented within this review could generally be attributed to one of two classifications, namely opportunistic/supplementary feeding, and the identification of new or expansion of current feeding grounds. We discuss the potential drivers of this behaviour, and the management challenges it presents.

### 4.1. Feeding Behaviour Overview

The feeding behaviour identified within these reviews spanned multiple phases of the migratory cycle and was facilitated by flexible foraging strategies. Within migratory corridors, opportunistic feeding occurred predominantly during the post-breeding, poleward migration. While migration towards breeding grounds is driven by the need to initiate reproduction, migration towards polar regions typically involves slower travel and extended periods at stopover sites [97,98]. Having expended significant energy reserves during reproduction and fasting [6], migrating individuals have an opportunity to offset their energetic deficit by exploiting opportunistic prey encountered before reaching polar feeding grounds. Some individuals actively anticipated or followed feeding opportunities away from traditional migratory corridors to target areas of high productivity [41]. Whereas others opportunistically exploited localised prey availability, such as when encountering and joining multi-species feeding aggregations [24]. Feeding also occurred beyond the core feeding season, with some individuals extending their foraging period by remaining longer at feeding grounds or by feeding at stopover sites encountered early in the subsequent migration [99].

Reports of summer feeding in mid-latitudes, when HWs would typically occupy high-latitude feeding grounds, are commonly suggested to represent the establishment of new, or expansion of existing feeding areas. For some populations, mid-latitude feeding regions have only been documented within the last few decades, such as the Magellan Strait in Chile, which was recognised in the early 2000s [16,100,101]. More recently, behavioural assessments have identified novel foraging areas within coastal estuaries, such as San Francisco Bay, where significant time was dedicated to feeding and milling, compared to surface active behaviours [77]. Similarly, a region off northern Patagonia has been consistently utilised for both foraging and nursing during the peak HW presence in the austral summer and autumn, suggesting a segregated feeding subpopulation [65]. Distinct cohorts within other HW populations exhibit mid-latitude summer residency, such as off the west coast of South Africa where a small subpopulation remains to exploit dense euphausiid assemblages [53]. Acoustic monitoring has also recorded HW song in this area during the austral summer, coinciding with the presence of super-group feeding formations, indicating that this region may serve dual purposes [57]. The NYB, with its proximity to more northern feeding grounds, such as the Gulf of Maine, is often considered an extension of this feeding area [102]. Its year-round use suggests it may function both as a feeding ground and a supplementary winter foraging area [103,104].

Foraging within lower-latitude breeding grounds is typically highly opportunistic, with most records involving only a few isolated individuals. Such isolated feeding events have been documented across multiple breeding regions, including the West Indies [90], Brazil [61], Hawaii [75], and Costa Rica [73]. Larger-scale feeding events have been recorded in breeding areas, such as aggregations of up to 50 individuals observed feeding concurrently in Mexican breeding waters, demonstrating the behavioural plasticity of HW foraging strategies.

### 4.2. Behavioural Drivers

Feeding along migratory corridors likely signifies an adaptive strategy, particularly by juvenile and non-breeding individuals, to prioritise energy replenishment over reproduction by foregoing the remaining migration to breeding grounds. Off the eastern United States, particularly in waters of the mid-Atlantic, many juveniles have been reported feeding during the winter breeding season, when they would typically be at lower latitudes [87,88,89]. A similar pattern has been observed off Brazil, where most feeding individuals were juveniles. Juvenile HWs are more vulnerable to nutritional stress during migration than other age classes, due to the energetic costs associated with growth and maturation [105,106]. As they are not yet sexually mature, juveniles may forego breeding and instead exploit productive habitats along migratory routes. These regions may represent more than opportunistic foraging sites, instead playing a crucial role in sustaining juveniles that may be unable to complete the full migration to primary feeding grounds. [59,62,86]. With growing populations and increasing environmental pressures, the importance of these areas to juvenile whales may become increasingly evident.

The need to supplement energy reserves may reflect continued population recovery from whaling, potentially leading to increased intra- and interspecific competition. As populations approach carrying capacity, intensified intraspecific competition may result in nutritional stress and difficulty in meeting an individual’s annual energetic requirements [107]. The foraging flexibility of HWs, particularly their capacity to switch between prey sources [108,109], likely supports their ability to exploit emerging foraging grounds. As reported in this review, the versatile feeding behaviour of HWs has been recorded in both the previously unrecognised importance of fish to a krill-dominated diet [40], and identification of new prey species for some populations [59,60]. The ability of HWs to act as a generalist species has likely driven their recovery from whaling while enhancing their overall resilience to changing ecosystems.

### 4.3. Environmental Drivers

Variable environmental conditions within migratory and breeding habitats heavily influence HW foraging behaviour. Most publications reported on instances of opportunistic feeding in migratory or breeding areas, where elevated primary and secondary productivity increased prey availability. Within breeding grounds, including waters off Tanzania, the Dominican Republic, Hawaii, Brazil, Ecuador, Nicaragua, and Costa Rica, feeding is generally reported as opportunistic, occurring in response to periods of high prey availability experienced in these regions during winter.

Many regions identified in this review are characterised by oceanographic features that enhance productivity and prey availability, including off eastern Australia (East Australian Current), South Africa (Benguela Upwelling System), South America (Humboldt Current), Central America (Costa Rica Dome) and Western US (California Frontal System) [110]. These systems support highly productive pelagic and coastal ecosystems and are likely associated with elevated feeding activity, as whales adjust migratory timing or routes to coincide with peaks in productivity [19]. In some regions, individuals remain well into the typical summer feeding season; for example, temperate feeding whales have been documented in Australian waters [21], and year-round presence has been reported off Mexico [74].

Substantial feeding activity, including large super-group aggregations, has been documented along the western coast of South Africa [27,28]. This region is influenced by the Benguela Upwelling System, which drives high primary and secondary productivity during the austral spring and summer and supports high prey availability [111]. Since 2011, these super-groups have been associated with environmental drivers such as phytoplankton blooms and reduced water export, and are likely influenced by population growth, shifts in prey availability, or both [26]. Similarly, the Humboldt Current System sustains high productivity along western South America [112], with regions such as Mejilones Bay or the Gulf of Corcovado along the Chilean coastline, seeing individuals return in consecutive years, suggesting persistent or expanding feeding habitat along this coastline [64,65]. These productive migratory regions may play an increasingly important role for some populations, particularly as variability in prey availability at high latitude feeding grounds drives shifts in feeding behaviour across migratory ranges [113,114].

### 4.4. Data Considerations

Data collection methods varied widely among publications, and many did not explicitly focus on recording feeding behaviour. Indirect indications of feeding, such as those derived from acoustic detection, stable isotope or fatty acid signatures, and prey in stomach contents, carry a higher potential for inaccurately assigning feeding location compared to direct observations. Additionally, ARS behaviour derived from satellite telemetry can be misclassified as foraging, as resting produces similar movement signatures. The observed bias in publications’ sampling effort towards nearshore regions potentially skewed results towards feeding in coastal areas and limited the representation of feeding in the high seas. While likely attributable to economic and logistical constraints, this is a prevalent issue across many facets of cetacean research [115]. Satellite tagging studies have the potential to increase the spatial coverage of studies with ARS behaviours detected in the open ocean through temperate regions [20].

Ambiguous terminology surrounding this feeding behaviour presented a challenge in both retrieving relevant publications and reporting on the prevalence of the behaviour. Whilst some specific terms, such as supplementary or opportunistic feeding, are increasingly used in literature, many of the publications included did not employ a specific term to describe the feeding behaviour reported. Additionally, habitat use within a region is not always well defined, meaning that separate studies focusing on the same locations may draw different conclusions about how those areas are classified. For instance, HW feeding areas along the US east coast are sometimes referred to as both feeding grounds and supplementary feeding areas, including off the coast of Virginia [86,89] and within the NYB [79,80,82]. Other terms used to describe this behaviour include winter foraging [59,88], or migratory stopover [43,47,94]. Older literature that did not explicitly define this behaviour may have been missed in initial searches and identified only through reference lists of included publications. Restricting inclusion to English-language publications may also have limited representation from some regions, particularly Asia. Considering these factors, feeding along migration or within breeding grounds is likely to occur more frequently than is reported in this paper. We propose clearer terminology for feeding outside polar or sub-polar feeding grounds, including during migration and within breeding areas. Although supplementary feeding is commonly used, terms such as “temperate feeding” or “flexible foraging” may be more appropriate.

### 4.5. Management Considerations

Polar ecosystems have been heavily impacted by climate change, including impacts to sea ice cover and ocean circulation [116,117], with further change projected [118]. These shifts directly affect high-latitude feeding grounds and may indirectly alter migration timing [119], and prey availability. Krill biomass and distribution are particularly threatened by climatic changes [120,121]. Prey availability and abundance are limiting factors for capital breeding HWs, and can impact both the body condition [122] and reproductive rates of individuals [123,124]. Declines in reproductive success and individual survival have been recorded for HWs corresponding to ecosystem shifts in their polar or sub-polar feeding grounds [125,126]. Mid-latitude regions where feeding occurs may be at risk of increased variability from climatic changes [127,128]. There is considerable uncertainty regarding how upwelling systems will evolve with climate change, and effects will likely be geographically dependent and highly variable [129]. Productivity may increase in some areas [130], yet it is uncertain whether this will result in increased plankton biomass and consequently feeding opportunities for migratory species [131].

The prevalence of feeding behaviour away from polar or sub-polar feeding grounds has implications for habitat protection and reduction of anthropogenic impacts on HWs and other capital breeding marine mammals. This review provides a consolidated overview for HWs, demonstrating that feeding can occur in most breeding or migratory areas when conditions are suitable. This suggests that the concept of distinct breeding, migrating, and feeding grounds is a simplified understanding of baleen whale ecology, and decision-makers must consider entire migratory corridors, including supplementary feeding locations, rather than just the termini, in species protection plans and ecosystem management.

Current protection measures in some of the feeding regions identified in this study are limited. In Australian waters, cetaceans face significant risks from entanglements and ship strikes [132], yet dedicated Marine Protected Areas (MPAs) cover only a small proportion of state waters in regions where HW feeding has occurred [133,134,135]. In Australian waters, Biologically Important Areas (BIAs) identify regions where protected species aggregate to perform biologically important behaviour. While BIAs are designated for HW breeding, resting, and migration, including coverage of the foraging regions along the NSW coastline, they do not carry direct legal protection and offer limited recognition of other opportunistic feeding sites [136]. Although MPAs encompass a portion of South Africa’s ocean territory [137], overlap with cetacean habitat is limited [138], and their effectiveness for highly mobile species is uncertain due to the significant distances they cover throughout migration [139]. In the NYB, Seasonal Management Areas (SMAs) impose vessel speed restrictions on large vessels during the peak occurrence of northern right whales. These SMAs are effective at protecting northern right whales from ship strikes [140], yet offer limited protection for other species that fall outside of their spatial and temporal bounds [141], including HWs [83,142].

As ongoing climatic changes continue to alter feeding conditions in high-latitude areas [116,117], MPAs alone may be insufficient within spatial or ecosystem-based management frameworks. HWs occupy extensive geographical ranges throughout their annual migrations, relying on diverse ocean habitats to mate, give birth, rest, socialise, and feed. Consequently, small, isolated MPAs focusing on a single region or ecosystem are often insufficient to protect these species across their critical ocean habitats [143]. The concept of “blue corridors” [144] has been proposed as ecologically important interconnected areas that marine megafauna utilise for survival as they move within these habitats. The areas recognised in this study as most important for feeding are highly dynamic and can change over time [145]. Effective dynamic ocean management requires the creation of connected, representative networks of MPAs [146] that protect whales not just at the terminus of their migratory pathways, but within all critical habitat areas. Additionally, the implementation of dynamic management tools, such as mobile MPAs, may help solve this issue [147]. By protecting these various feeding regions, as well as the terminals of migration, we can try to safeguard food sources for baleen whales and ensure their survival under future climate change.

## 5. Conclusions

This systematic review demonstrates that HW feeding along migratory routes and in breeding grounds is a widespread global phenomenon driven by behavioural plasticity and localised oceanographic productivity. This behaviour challenges the traditional view that strictly separates high-latitude feeding from low-latitude breeding, revealing instead that whales frequently utilise alternative foraging strategies. As climate change and population growth are expected to alter foraging dynamics in high-latitude ecosystems, these mid- and low-latitude opportunistic feeding events and expanding temperate foraging grounds are likely to become increasingly important for the species’ resilience.

These emerging feeding zones could potentially overlap with human activity; therefore, effective protection requires safeguarding entire migratory ranges rather than just the endpoints. This highlights the need to shift conservation frameworks from isolated MPAs toward interconnected and dynamic mobile management tools. Standardising research terminology around flexible foraging and expanding data collection beyond coastal zones will be essential next steps to properly manage and mitigate anthropogenic risks in these critical habitats.

## Figures and Tables

**Table 1:** Methods of data collection utilised and the data type analysed within the publications included in this literature review, and the number of studies that employed them.

Method of Data Collection	No. of Studies	Data Analysed	No. of Studies
Boat-based observational surveys	36	Observational data	53
Necropsies of stranded specimens	15	Identification photos	19
Tourism operators	15	Location data from satellite tags	16
Biopsy sampling	14	Skin samples	12
Satellite tags	11	Environmental data	11
Land-based observational surveys	11	Prey samples	8
Opportunistic sightings	8	Body measurements	7
Aerial surveys	8	Stomach contents	5
Acoustic detection	6	Audio data	5
Anecdotal data (citizen science, social media)	5	Baleen samples	3
UAV footage	6	Blubber/biopsy samples	4
		Faecal samples	2

## Data Availability

Dataset available on request from the authors.

## Supplementary Materials

The following supporting information can be downloaded at: https://www.mdpi.com/article/10.3390/ani16172799/s1, Material S1: The recognised general breeding and feeding grounds of humpback whale populations worldwide; Material S2: List of included publications; Material S3: Recorded prey species consumed by humpback whale populations, compiled from published literature. References [148,149,150,151,152,153,154,155,156,157,158,159,160,161] are cited in the supplementary materials.
