# Supplementary material for: Humpback Whales (Megaptera novaeangliae) Feeding Along Migration Routes and Within Breeding Grounds—A Review
Source: Animals (Basel). 2026 Sep 6;16(17):2799. doi: 10.3390/ani16172799 (PMC13565171; doi:10.3390/ani16172799)
Supplement: Supplementary file 1 [file animals-16-02799-s001.zip › Supplementary Material S1_HW.pdf]

Supplementary Material S1: The recognised general breeding and feeding grounds of humpback whale populations worldwide

**Northern Hemisphere**	**Western North Atlantic**<br>Population part of the West Indies DPS - breeding range includes the Atlantic margin of the Antilles from Cuba to northern Venezuela [148]. Four feeding aggregations - the Gulf of Maine, West Greenland, eastern Canada and the eastern North Atlantic [149].<br>Some appear to also use feeding grounds in the central (Iceland) and eastern (Norway) North Atlantic [148].
	**Eastern North Atlantic**<br>Feeding grounds believed to be off Greenland, Iceland, and Norway [150].<br>Separate feeding aggregations may be present from western Iceland and the Greenland Sea, and another extending from eastern Iceland into the Barents Sea [149].<br>Rare sightings off the Murman coast in northwestern Russia [151].<br>The Cape Verde Islands appear to be a breeding ground for this species [152], with none of these individuals sighted at other North Atlantic summering or wintering grounds [153].<br>Appear to be common feeding grounds off Iceland and in the Barents Sea [153].
	**Eastern North Pacific - California/Oregon/ Washington-Mexico**<br>Population of the Central America DPS, breed along the Pacific coast of Costa Rica, Panama, Guatemala, El Salvador, Honduras and Nicaragua. Feed almost exclusively along offshore of California and Oregon, a few individuals feed at the northern Washington –southern British Columbia feeding grounds [148].<br>Whales identified from the mainland coast of Mexico, Baja California Peninsula and Revillagigedo Archipelago have been matched to 5 feeding grounds: western Gulf of Alaska, Prince William Sound, southeastern Alaska, British Columbia, California-Oregon-Washington region [154].
	**Mexico offshore**<br>The Mexican DPS whales breed along mainland Mexico, the Baja California Peninsula and the Revillagigedos Islands [155].<br>Matched to 5 feeding grounds: western Gulf of Alaska, Prince William Sound, southeastern Alaska, British Columbia, California-Oregon-Washington region [154].<br>Also occasionally encountered in the Gulf of Anadyr and off the Commander Islands [155].<br>Historical feeding grounds thought to be off the Aleutian Islands and/or the Bering Sea [154].
	**Central North Pacific**<br>The Hawaii DPS whales breed within the main Hawaiian Islands.<br>Feed in most of the North Pacific feeding grounds, most feed off Southeast Alaska and Northern British Columbia, as well as the northern Gulf of Alaska and Bering Sea feeding grounds [148].<br>Sometimes seen off Kamchatka, the Commander Islands, the Kuril Islands and the Gulf of Anadyr [155].
	**Western North Pacific**<br>The Okinawa/Philippines DPS whales breed Okinawa and Ogasawara, Japan and the Babuyan Islands in the northern Philippines [148], [156].<br>They feed primarily off the Russian coast, off Kamchatka [148].<br>There is thought to be a second West Pacific DPS that feed near the Aleutian Islands and in waters of the Russian Far East, but are not linked to a known breeding population [148], [155].
	**Arabian Sea**<br>The Arabian Sea DPS whales breed and feed along the coast of Oman, in the Gulf of Oman and Persian Gulf [157] with historical records from the eastern Arabian Sea along the coasts of Pakistan and India, so may reside there too [148].<br>The population is small and is non-migratory resident in the Northern Indian Ocean [158].<br>Have been observed bubble-net feeding [150].

**Southern Hemisphere**	Feeding stocks I-VI. Limits are:<br>I = 120°W–60°W<br>II = 60°W–0°<br>III = 0–70°E (IIIW = 0–35°E and IIIE = 35°E–70°E)<br>IV = 70°E–130°E (IVW = 70°E–100°E and IVE = 100°E–130°E)<br>V = 130°E–170°E (VW = 130°E–150°E and VE = 150°E–170°E)<br>VI = 170°E–120°W (VIW = 170°W–155°W and VIE = 155°W–120°W).<br>Separated into breeding stocks A to G
	**Breeding stock A:**<br>Breed along east coast of South America, Brazil, feed off South Georgia and the South Sandwich Islands [148].
	**Breeding stock B:**<br>Breed along west coast of Africa (B1 and B2), Gabon, Namibia and South Africa western coast, feed offshore of west South Africa, south of 18°S and in the Southern Ocean beneath west South Africa [148], [159].
	**Breeding stock C:**<br>Breed along east coast of Africa and western Indian Ocean, Mozambique and southern Tanzania, Comoros Archipelago, Madagascar, Mascarene Islands of Mauritius and Reunion (C1, C2, C3, C4).<br>Feed in the Southern Ocean, areas not well defined but are believed to include multiple localities to the west and east of the region bounded by 10°W – 60°E [159]
	**Breeding stock D:**<br>Breed along west Australia, feeding areas in the Antarctic, primarily between 80°E and 110°E [148], [159]
	**Breeding stock E:**<br>Breed along east Australia (E1), New Caledonia (E2) and Tonga (E3), migrate to Antarctic feeding areas ranging from 100°E to 180°E, [148], but concentrated mostly between 120°E and 170°W [159]
	**Breeding stock F:**<br>The Oceania DPS whales breed in the South Pacific Islands between ~160°E (west of New Caledonia) to ~120°W (east of French Polynesia), predominately Cook Islands (F1) and French Polynesia (F2) [159], they feed in Antarctic waters 170°W and 110°W [159]
	**Breeding stock G:**<br>The Southeastern Pacific DPS whales that breed along the Pacific coasts of Panama to northern Peru (9°N-6°S), main wintering areas in Colombia. Feeding grounds concentrated in the Chilean Magellan Straits and the western Antarctic Peninsula [148], 110°W and 50°W [159]

References:

3. Mikhalev, Y. A. Humpback whales Megaptera novaeangliae in the Arabian Sea. *Marine Ecology Progress Series* **1997**, 149, 13-21. doi:https://doi.org/10.3354/meps149013.

148. Bettridge, S. O. M.; Baker, C. S.; Barlow, J.; Clapham, P.; Ford, M. J.; Gouveia, D.; Mattila, D. K.; Pace, R. M.; Rosel, P. E.; Silber, G. K.; et al. Status review of the humpback whale (Megaptera novaeangliae) under the Endangered Species Act; 2015.

149. Stevick, P. T.; Allen, J.; Clapham, P. J.; Katona, S. K.; Larsen, F.; Lien, J.; Mattila, D. K.; Palsbøll, P. J.; Sears, R.; Sigurjónsson, J.; et al. Population spatial structuring on the feeding grounds in North Atlantic humpback whales (Megaptera novaeangliae). *Journal of Zoology* **2006**, 270, 244-255, doi:https://doi.org/10.1111/j.1469-7998.2006.00128.x.

150. Clapham, P. J. Humpback Whale: Megaptera novaeangliae. In Encyclopedia of Marine Mammals (3rd Edition), Würsig, B., Thewissen, J.G.M., Kovacs, K.M., Eds.; Academic Press: **2018**; pp. 489-492. doi:https://doi.org/10.1016/B978-0-12-804327-1.00154-0

151. Peres Dos Santos, R.; Martins, R.; Chaiko, A.; Cheeseman, T.; Jones, L. S.; Wenzel, F. W. First humpback whale (Megaptera novaeangliae) re-sighting between Azorean waters and the Barents Sea (Murman coast, northwestern Russia). *Polar Biology* **2022**, 45, 523-527, doi:https://doi.org/10.1007/s00300-021-02998-y.

152. Wenzel, F.; Allen, J.; Berrow, S.; Hazevoet, C.; Jann, B.; Seton, R.; Steiner, L.; Stevick, P.; Lopez-Suarez, P.; Whooley, P. Current Knowledge on the Distribution and Relative Abundance of Humpback Whales (Megaptera novaeangliae) off the Cape Verde Islands, Eastern North Atlantic. *Aquatic Mammals* **2009**, 35, 502-510, doi:https://doi.org/10.1578/AM.35.4.2009.502

153. Ryan, C.; Mchugh, B.; Boyle, B.; Mcgovern, E.; Bérubé, M.; Lopez-Suárez, P.; Elfes, C. T.; Boyd, D. T.; Ylitalo, G. M.; Van Blaricom, G. R. Levels of persistent organic pollutants in eastern North Atlantic humpback whales. *Endangered Species Research* **2013**, 22, 213-223. doi:https://doi.org/10.3354/esr00545

154. Urbán R., J.; Jaramillo L., A.; Aguayo L., A.; Ladrón De Guevara P., P.; Salinas Z., M.; Alvarez F., C.; Medrano G., L.; Jacobsen, J. K.; Balcomb, K. C.; Claridge, D. E.; et al. Migratory destinations of humpback whales wintering in the Mexican Pacific. *Journal of Cetacean Research and Management* **2016**, 2, 101-110, doi: https://doi.org/10.47536/jcrm.v2i2.493.

155. Titova, O. V.; Fedutin, I. D.; Filatova, O. A.; Antipin, M. A.; Burdin, A. M.; Hoyt, E. The Characteristics of the Feeding Aggregation Formed by Humpback Whales, Megaptera novaeangliae (Borowski, 1781), in Senyavin Strait, off the Eastern Chukotka Coast, According to Photo-Identification Data. *Russian Journal of Marine Biology* **2020**, 46, 330-337, doi: https://doi.org/10.1134/S1063074020050119.

156. Silberg, J. N.; Acebes, J. M. V.; Burdin, A. M.; Mamaev, E. G.; Dolan, K. C.; Layusa, C. A.; Aca, E. Q. New insight into migration patterns of western North Pacific humpback whales between the Babuyan Islands, Philippines and the Commander Islands, Russia. *Journal of Cetacean Research and Management* **2013**, 13, 53-57. doi:https://doi.org/10.47536/jcrm.v13i1.555

157. Dakhteh, S.; Ranjbar, S.; Moazeni, M.; Mohsenian, N.; Delshab, H. The Persian Gulf is part of the habitual range of the Arabian Sea Humpback whale population. *Journal of Marine Biology & Oceanography* **2017**, 3, 2.

158. Minton, G.; Findlay, K.; Ersts, P.; Rosenbaum, H.; Berggren, P.; Baldwin, R. Seasonal distribution, abundance, habitat use and population identity of humpback whales in Oman. *Journal of Cetacean Research and Management* **2011**, 3, 185-198, doi: https://doi.org/10.47536/jcrm.vi3.329.

159. Seyboth, E.; Meynecke, J.-O.; De Bie, J.; Roychoudhury, A.; Findlay, K. A review of post-whaling abundance, trends, changes in distribution and migration patterns, and supplementary feeding of Southern Hemisphere humpback whales. *Frontiers in Marine Science* **2023**, 10, 997491. doi:https://doi.org/10.3389/fmars.2023.997491
