# Supplementary material for: Humpback Whales (Megaptera novaeangliae) Feeding Along Migration Routes and Within Breeding Grounds—A Review
Source: Animals (Basel). 2026 Sep 6;16(17):2799. doi: 10.3390/ani16172799 (PMC13565171; doi:10.3390/ani16172799)
Supplement: Supplementary file 1 [file animals-16-02799-s001.zip › Supplementary Material S3_HW.pdf]

Supplementary Material 3: Recorded prey species consumed by humpback whale populations, compiled from published literature.

**HW Population**	**Prey species recorded**	**Source**
North Atlantic	Northern krill (*Meganyctiphanes norvegica*)<br>Herring (*Clupea harengus*)	[19],[96]
Western North Atlantic	Atlantic menhaden (*Brevoortia tyrannus*)<br>Sand lance (*Ammodytes spp.*)<br>Banded drum (*Larimus fasciatus*)<br>Atlantic croaker (*Micropogonias undulatus*)<br>Spot (*Leiostomus xanthurus*)<br>Weakfish (*Cynoscion regalis*)<br>Silver seatrout (*Cynoscion nothus*)	[82],[85],[87]
Eastern North Pacific	Euphausiids (*Nyctiphanes simplex*)<br>Northern Anchovy (*Engraulis mordax*)<br>Pacific anchovies (*Cetengraulis mysticetus*)	[69],[70],[77]
Breeding Stock A	Sergestid shrimp (*Peisos petrunkevitch*) *<br>Aviu shrimp (*Acetes americanus*) *	[59],[60]
Breeding Stock B	Cape mantis shrimp (*Pterygosquilla armata capensis*)<br>Krill (*Euphausia lucens*)	[27],[55],[56]
Breeding stock E1	Australian krill (*Nyctiphanes australis*)<br>Jack Mackerel (*Trachurus declivis*)<br>Redbait (*Emmelichthys nitidus*)<br>Pilchards (*Sardinops neopilchardus*)	[18],[42],[43],<br>[160],[161]
Breeding stock G	Peruvian anchovy (*Engraulis ringens*)<br>Krill (*Euphausia valentinii*)<br>Squat lobster (*Munida spp. (M. subrugosa/gregaria*))	[64],[65]

* New prey species identified

References

18. Stamation, K. A.; Croft, D. B.; Shaughnessy, P. D.; Waples, K. A. Observations of Humpback Whales (*Megaptera novaeangliae)* Feeding During Their Southward Migration Along the Coast of Southeastern New South Wales, Australia: Identification of a Possible Supplemental Feeding Ground. *Aquatic Mammals* **2007**, *33*, 165-174, doi:https://doi.org/10.1578/AM.33.2.2007.165.
19. Visser, F.; Hartman, K. L.; Pierce, G. J.; Valavanis, V. D.; Huisman, J. Timing of migratory baleen whales at the Azores in relation to the North Atlantic spring bloom. *Marine Ecology Progress Series* **2011**, 440, 267-279, doi:https://doi.org/10.3354/meps09349.
27. Findlay, K. P.; Seakamela, S. M.; Meyer, M. A.; Kirkman, S. P.; Barendse, J.; Cade, D. E.; Hurwitz, D.; Kennedy, A. S.; Kotze, P. G. H.; Mccue, S. A.; et al. Humpback whale "super-groups" – A novel low-latitude feeding behaviour of Southern Hemisphere humpback whales (*Megaptera novaeangliae)* in the Benguela Upwelling System. *PLoS ONE* **2017**, *12*, e0172002, doi:https://doi.org/10.1371/journal.pone.0172002.
42. Owen, K.; Kavanagh, A. S.; Warren, J. D.; Noad, M. J.; Donnelly, D.; Goldizen, A. W.; Dunlop, R. A. Potential energy gain by whales outside of the Antarctic: prey preferences and consumption rates of migrating humpback whales (*Megaptera novaeangliae*). *Polar Biology* **2017**, *40*, 277-289, doi:https://doi.org/10.1007/s00300-016-1951-9.
43. Owen, K.; Warren, J. D.; Noad, M. J.; Donnelly, D.; Goldizen, A. W.; Dunlop, R. A. Effect of prey type on the fine-scale feeding behaviour of migrating east Australian humpback whales. *Marine Ecology Progress Series* **2015**, *541*, 231-244, doi:https://doi.org/10.3354/meps11551
55. Barendse, J.; Best, P. B.; Thornton, M.; Pomilla, C.; Carvalho, I.; Rosenbaum, H. C. Migration redefined? Seasonality, movements and group composition of humpback whales *Megaptera novaeangliae* off the west coast of South Africa. *African Journal of Marine Science* **2010**, *32*, 1-22, doi:https://doi.org/10.2989/18142321003714203.
56. Findlay, K. P.; Best, P. B. Summer Incidence of Humpback Whales on the West-Coast of South-Africa. *South Afr. J. Mar. Sci.-Suid-Afr. Tydsk. Seewetens.* **1995**, *15*, 279-282, doi:https://doi.org/10.2989/02577619509504851.
59. Bortolotto, G. A.; Kolesnikovas, C. K. M.; Freire, A. S.; Simões-Lopes, P. C. Young humpback whale *Megaptera novaeangliae* feeding in Santa Catarina coastal waters, Southern Brazil, and a ship strike report. *Marine Biodiversity Records* **2016**, *9*, 29, doi:https://doi.org/10.1186/s41200-016-0043-4.
60. Danilewicz, D.; Tavares, M.; Moreno, I. B.; Ott, P. H.; Trigo, C. C. Evidence of feeding by the humpback whale (*Megaptera novaeangliae*) in mid-latitude waters of the western South Atlantic. *Marine Biodiversity Records* **2009**, *2*, E88, doi:https://doi.org/10.1017/S1755267209000943.
64. Cegarra, A. M. G.; Castro, C.; Van Waerebeek, K. Feeding of humpback whales in low latitudes of the Southeast Pacific Ocean. *Neotrop. Biodivers.* **2021**, *7*, 421-430, doi:https://doi.org/10.1080/23766808.2021.1971041.
65. Hucke-Gaete, R.; Haro, D.; Torres-Florez, J. P.; Montecinos, Y.; Viddi, F.; Bedriñana-Romano, L.; Nery, M. F.; Ruiz, J. A historical feeding ground for humpback whales in the eastern South Pacific revisited: the case of northern Patagonia, Chile. *Aquatic Conservation: Marine and Freshwater Ecosystems* **2013**, *23*, 858-867, doi:https://doi.org/10.1002/aqc.2343.
69. Frisch-Jordán, A.; Ransome, N.; Aranda-Mena, O.; Romo-Sirvent, F. Intensive feeding of humpback whales (*Megaptera novaeangliae*) in the breeding ground of Banderas Bay, Mexico. *Latin American Journal of Aquatic Mammals* **2019**, *14*, 27-33, doi:https://doi.org/10.5597/00251.
70. Gendron, D.; Urban R, J. Evidence of feeding by humpback whales (*Megaptera novaeangliae*) in the Baja California breeding ground, Mexico. *Marine Mammal Science* **1993**, *9*, 76-81, doi:https://doi.org/10.1111/j.1748-7692.1993.tb00428.x.

77. Markowitz, T. M.; Keener, W.; Webber, M. A.; Payne, A. R.; Lane, R. S.; Fahlbusch, J. A.; Calambokidis, J. New urban habitat for endangered humpback whales: San Francisco Bay. *Aquatic Conservation: Marine and Freshwater Ecosystems* **2024**, *34*, e4107, doi:https://doi.org/10.1002/aqc.4107.
82. Brown, D. M.; Robbins, J.; Sieswerda, P. L.; Schoelkopf, R.; Parsons, E. C. M. Humpback whale (*Megaptera novaeangliae*) sightings in the New York-New Jersey Harbor Estuary. *Marine Mammal Science* **2018**, *34*, 250-257, doi:https://doi.org/10.1111/mms.12450.
85. Aschettino, J. M.; Engelhaupt, D. T.; Engelhaupt, A. G.; Dimatteo, A.; Pusser, T.; Richlen, M. F.; Bell, J. T. Satellite Telemetry Reveals Spatial Overlap Between Vessel High-Traffic Areas and Humpback Whales (*Megaptera novaeangliae*) Near the Mouth of the Chesapeake Bay. *Frontiers in Marine Science* **2020**, *7*, 121, doi:https://doi.org/10.3389/fmars.2020.00121.
87. Laerm, J.; Wenzel, F.; Craddock, J. E.; Weinand, D.; Mcgurk, J.; Harris, M. J.; Early, G. A.; Mead, J. G.; Potter, C. W.; Barros, N. B. New Prey Species for Northwestern Atlantic Humpback Whales. *Marine Mammal Science* **1997**, *13*, 705-711, doi:https://doi.org/10.1111/j.1748-7692.1997.tb00094.x
96. Jourdain, E.; Vongraven, D. Humpback whale (*Megaptera novaeangliae*) and killer whale (*Orcinus orca*) feeding aggregations for foraging on herring (*Clupea harengus*) in Northern Norway. *Mammalian Biology* **2017**, *86*, 27-32, doi:https://doi.org/10.1016/j.mambio.2017.03.006.
160. Gill, P.; Evans, K.; Wapstra, H. Feeding by humpback whales in Tasmanian waters. *Records of the Queen Victoria Museum* **1999**, *107*.
161. Owen, K.; Thompson, R. M.; Donnelly, D.; Noad, M.; Bury, S. J.; Pinkerton, M. H.; Dunlop, R. Southern Ocean humpback whale trophic ecology. II. Influence of fasting and opportunistic feeding on skin stable isotope values of migrating whales. *Marine Ecology Progress Series* **2024**, *734*, 157-171, doi:https://doi.org/10.3354/meps14539.
